# The duration of beta‐blocker therapy and outcomes in patients without heart failure or left ventricular systolic dysfunction after acute myocardial infarction: A multicenter prospective cohort study

**DOI:** 10.1002/clc.23807

**Published:** 2022-03-04

**Authors:** Xue‐Song Wen, Rui Luo, Jie Liu, Zhi‐Qiang Liu, Han‐Wen Zhang, Wei‐Wei Hu, Qin Duan, Shu Qin, Jun Xiao, Dong‐Ying Zhang

**Affiliations:** ^1^ Department of Cardiovascular Medicine The First Affiliated Hospital of Chongqing Medical University Chongqing China; ^2^ Department of Cardiovascular Medicine The First Branch of the First Affiliated Hospital of Chongqing Medical University Chongqing China; ^3^ Department of Cardiovascular Medicine Chongqing University Center Hospital Chongqing China

**Keywords:** acute myocardial infarction, beta‐blockers, heart failure, left ventricular ejection fraction, probability

## Abstract

**Background:**

The duration of beta‐blocker therapy in patients without heart failure (HF) or left ventricular systolic dysfunction after acute myocardial infarction (AMI) is unclear.

**Hypothesis:**

Continuous beta‐blocker therapy is associated with an improved prognosis.

**Methods:**

This is a prospective, multicenter, cohort study. One thousand four hundred and eighty‐three patients eventually met the inclusion criteria. The study groups included the continuous beta‐blocker therapy group (lasted ≥6 months) and the discontinuous beta‐blocker therapy group (consisting of the no‐beta‐blocker therapy group and the beta‐blocker therapy <6 months group). The inverse probability treatment weighting was used to control confounding factors. The study tried to learn the role of continuous beta‐blocker therapy on outcomes. The median duration of follow‐up was 13.0 months. The primary outcomes were cardiac death and major adverse cardiovascular events (MACE). The secondary outcomes were all‐cause death, stroke, unstable angina, rehospitalization for HF, and recurrent myocardial infarction (MI).

**Results:**

Compared with discontinuous beta‐blocker therapy, continuous beta‐blocker therapy was associated with a reduced risk of unstable angina, recurrent MI, and MACE (hazard ratio [HR]: 0.51; 95% CI: 0.32–0.82; *p* = 0.006); but this association was not available for cardiac death (HR: 0.57; 95% CI: 0.24–1.36; *p* = 0.206). When compared to the subgroups of no‐beta‐blocker therapy and beta‐blocker therapy <6 months, respectively, continuous beta‐blocker therapy was still observed to be associated with a reduced risk of unstable angina, recurrent MI, and MACE.

**Conclusions:**

Continuous beta‐blocker therapy was associated with a reduced risk of unstable angina or recurrent MI or MACE in patients without HF or left ventricular systolic dysfunction after AMI.

**Abbreviations**
ACEI: angiotensin‐converting enzyme inhibitor
AMI: acute myocardial infarction
ARB: angiotensin receptor blocker
ARNI: angiotensin receptor enkephalin inhibitor
HF: heart failure
IPTW: inverse probability of treatment weighting
LVEF: left ventricular ejection fraction
MACE: major cardiovascular adverse events
PCI: percutaneous coronary intervention
STEMI: ST‐segment elevation myocardial infarction

## 1 INTRODUCTION

Some milestone studies such as the BHAT (The Beta‐blocker Heart Attack Trial), and the ISIS‐I (First International Study of Infarct Survival) had established that beta‐blockers can significantly reduce mortality in patients with myocardial infarction (MI) was published in the 1980s.^1^, ^2^, ^3^ The beta‐blockers then become a central component of pharmacological treatment for acute myocardial infarction (AMI). Subsequently, progress has been made in the treatment of MI and mortality has decreased remarkably thanks to the application of treatments such as percutaneous coronary intervention (PCI), antiplatelet drugs, and statins.^4^ Because of this, it is questionable whether beta‐blockers can still benefit AMI patients at a time when reperfusion treatment and secondary prevention therapy are widely available.

There are difficulties in the precise application of beta‐blockers in patients with AMI. Guidelines are inconsistent regarding the indication population for beta‐blocker therapy. Evidence demonstrates that beta‐blocker therapy is essential as a cornerstone in the treatment of AMI patients with reduced left ventricular systolic function (LVEF < 40%).^5^, ^6^ However, the efficacy of beta‐blockers in AMI patients with midrange/preserved left ventricular ejection fraction (LVEF ≥ 40%) is unclear.^7^ Also, Guidelines or consensus, with fewer recommendations for the duration of beta‐blocker therapy after AMI. 2012 ACCF recommends that beta‐blocker therapy be continued for 3 years in patients with the acute coronary syndrome who have a normal left ventricular function (LVEF > 40%).^8^ The latest ESC Guidelines for the management of AMI in patients presenting with ST‐segment elevation do not give any recommendations in this respect.^5^ The Canadian Heart Research Centre recommends, based on consensus, patients with a mild‐moderate reduction of left ventricular function (LVEF ≥ 40%) who have undergone successful reperfusion, treatment discontinuation could be considered after 6 months.^9^ The benefits of early beta‐blocker therapy have been demonstrated,^10^, ^11^ whereas few studies have been conducted on the duration of beta‐blocker therapy, with more attention focused on the impact of long‐term beta‐blocker therapy on outcomes.

The purpose of this study was to learn the effect of continuous beta‐blockers therapy (lasted ≥6 months) on AMI patients without heart failure (HF) or left ventricular systolic dysfunction.

## 2 METHODS

### 2.1 Study design and data collection

The study is a multicenter, prospective, cohort, observational registry project with clinicaltrials. gov identifier NCT04564365. We observed the Declaration of Helsinki guidelines. All study procedures were approved by the local ethics committee (approval number 2020‐607).

We enrolled patients hospitalized for AMI from five hospitals between April 2019 and April 2021. The baseline characteristics of the patients were collected through the medical record. The epidemiological data, risk factors, comorbidities, treatments, and prescribed medication information of the patients were recorded. During follow‐up, the information on patient survival status and hospitalization events was collected through telephone interviews and medical documents.

### 2.2 Population

**Figure 1:**
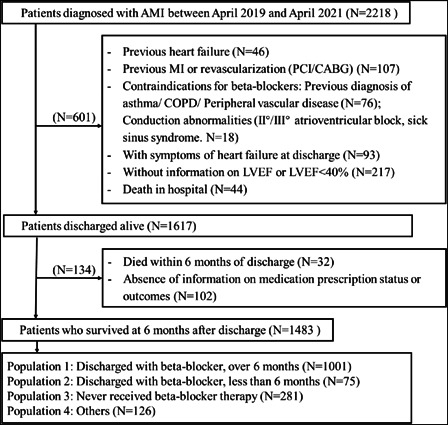
Flow diagram of patients recruitment. Others: Discharged without beta‐blocker therapy, restarted beta‐blocker therapy during the follow‐up. AMI, acute myocardial infarction; CABG, coronary artery bypass grafting; COPD, chronic obstructive pulmonary disease; LVEF, left ventricular systolic function; MI, myocardial infarction; PCI, percutaneous coronary intervention

Patients diagnosed with AMI from five hospitals were recruited consecutively from April 2019 to April 2021. This study initially enrolled 2218 patients with AMI. Patients with a history of HF (*N* = 46), AMI or reperfusion therapy (*N* = 107), patients with contraindications to beta‐blocker use (including chronic obstructive pulmonary disease, asthma, peripheral vascular disease, second‐degree/third‐degree atrioventricular block, and sick sinus node syndrome, *N* = 94), patients with symptoms of HF at discharge (*N* = 93), patients without information on LVEF or with LVEF < 40% (*N* = 217), and patients died in hospital (*N* = 44) were excluded from the study. In addition, 32 patients died within 6 months and 102 patients lacked information on medication prescriptions or lost interviews, all of whom were also excluded. Ultimately, 1483 patients were included. This study included two groups, the continuous beta‐blocker therapy group (*N* = 1001) and the discontinuous beta‐blocker therapy group (*N* = 356, consisting of the no‐beta‐blocker therapy group and the beta‐blocker therapy <6 months group; Figure 1).

### 2.3 Statistical analysis

Continuous variables were presented as mean ± standard deviation or median (interquartile range). Categorical variables were expressed as frequencies and percentages. Continuous variables were compared by using the independent samples *T*‐test and the Mann–Whitney *U*‐test. Categorical variables were tested by using the *χ*2 test and Fisher's exact *χ*2 test. The study was conducted with propensity score inverse probability treatment weighting (IPTW) to minimize confounders. The propensity score was estimated using a logistic regression model based on the clinical characteristics listed in Table 1 (except for the duration variable). The IPTW weighted Cox regression analyses were used to determine the associations between beta‐blockers and outcomes. Kaplan–Meier curves were used to assess prognostic differences between the groups, using log‐rank tests. The R Statistical Package, version 4.0.2 (R Development Team), and IBM SPSS Statistics 26.0 software (SPSS) were used for all statistical analyses. *p* (two‐tailed) value less than 0.05 was considered statistically significant.

**Table 1:** Clinical characteristics stratified by beta‐blockers therapy status

Variables	Continuous beta‐blocker therapy (*N* = 1001)	Discontinuous beta‐blocker therapy (*N* = 356)	*p*‐value^a^	Beta‐blocker therapy <6 months (*N* = 75)^b^	No‐beta‐blocker therapy (*N* = 281)^c^
*Baseline characteristics*					
Age, years	64.0 (54.0–72.0)	66.0 (56.5–74.0)	0.005	67.0 (59.0–74.5)*	65.0 (56.0–74.0)*
Age ≥75 years	175 (17.5%)	85 (23.9%)	0.010	19 (25.3%)	66 (23.5%)*
Male sex	786 (78.5%)	275 (77.2%)	0.654	58 (77.3%)	217 (77.2%)
*Risk factors—no, %*					
Hypertension	585 (58.4%)	169 (47.5%)	<0.001	39 (52.0%)	130 (46.3%)***
Diabetes mellitus	374 (37.4%)	120 (33.7%)	0.224	28 (37.3%)	92 (32.7%)
Hyperlipidemia	262 (26.2%)	81 (22.8%)	0.227	17 (22.7%)	64 (22.8%)
Cigarette smoking	610 (60.9%)	232 (65.2%)	0.162	41 (54.7%)	191 (68.0%)*
Family history of CAD	42 (4.2%)	18 (5.1%)	0.548	2 (2.7%)	16 (5.7%)
*Medical history—no, %*					
Previous CAD	48 (4.8%)	25 (7.0%)	0.131	8 (10.7%)	17 (6.0%)
Chronic kidney disease	64 (6.4%)	19 (5.3%)	0.522	3 (4.0%)	16 (5.7%)
Previous stroke or TIA	75 (7.5%)	27 (7.6%)	1.000	4 (5.3%)	23 (8.2%)
Atrial fibrillation	42 (4.2%)	19 (5.3%)	0.373	8 (10.7%)*	11 (3.9%)
Malignant tumor	18 (1.8%)	6 (1.7%)	1.000	1 (1.3%)	5 (1.8%)
*Myocardial infarction characteristics—no, %*					
STEMI	648 (64.7%)	231 (64.9%)	1.000	42 (56.0%)	189 (67.3%)
Anterior MI	396 (61.1%)	77 (33.3%)	<0.001	22 (52.4%)	55 (29.1%)***
Inferior/posterior MI	276 (42.6%)	157 (68.0%)	<0.001	22 (52.4%)	135 (71.4%)***
Other sites MI	106 (16.4%)	40 (17.3%)	0.758	9 (21.4%)	31 (16.4%)
Coronary angiography	949 (94.8%)	322 (90.4%)	0.005	70 (93.3%)	252 (89.7%)**
Thrombolytic therapy	77 (7.7%)	18 (18.9%)	0.115	3 (4.0%)	15 (5.3%)
PCI therapy	823 (82.2%)	252 (70.8%)	<0.001	60 (80.0%)	192 (68.3%)***
PCI within 72 h	432 (43.2%)	129 (36.2%)	0.024	26 (34.7%)	103 (36.7%)
Other PCI	385 (38.5%)	121 (34.0%)	0.142	34 (45.3%)	87 (31.0%)*
CABG	1 (0.1%)	0 (0%)	1.000	0 (0.0%)	0 (0.0%)
Timely reperfusion therapy	265 (26.5%)	80 (22.5%)	0.156	17 (22.7%)	63 (22.4%)
Total revascularization	832 (83.1%)	253 (71.1%)	<0.001	61 (81.3%)	192 (68.3%)***
*Presenting characteristics*					
LVEF (%)	57.0 (50.0–61.0)	57.0 (51.0–63.0)	0.025	56.0 (49.5–61.0)	58.0 (52.0–63.0)*
Cardiac aneurysm	47 (4.7%)	8 (2.2%)	0.043	3 (4.0%)	5 (1.8%)*
*Concomitant medications—no, %*					
Aspirin	945 (94.4%)	330 (92.7%)	0.246	68 (90.7%)	262 (93.2%)
Clopidogrel/ticagrelor	989 (98.8%)	349 (98.0%)	0.298	73 (97.3%)	276 (98.2%)
DAPT	948 (94.7%)	325 (91.3%)	0.029	66 (88.0%)*	259 (92.2%)
Statin	995 (99.4%)	348 (97.8%)	0.014	72 (96.0%)*	276 (98.2%)
ACEI/ARB/ARNI	726 (72.5%)	193 (54.2%)	<0.001	65 (69.3%)	141 (50.2%)***
Oral anticoagulant	42 (4.2%)	17 (4.8%)	0.651	5 (6.7%)	12 (4.3%)
SGLT2i/DPP4i/GLP1Ras	134 (13.4%)	41 (11.5%)	0.408	11 (14.7%)	30 (10.7%)
Diuretics	221 (22.1%)	62 (17.4%)	0.068	10 (13.3%)	52 (18.5%)
*Duration of beta‐blocker therapy, days*					
Duration	383.0 (276.0–517.0)	‐	‐	96.0 (39.0–141.0)	‐

Abbreviations: ACEI, angiotensin‐converting enzyme inhibitor; ARB, angiotensin receptor blocker; ARNI, angiotensin receptor enkephalin inhibitor; CABG, coronary artery bypass grafting; CAD, coronary atherosclerotic heart disease; DAPT, dual antiplatelet therapy; DPP4i, dipeptidyl peptidase −4 inhibitors; GLP1Ras, glucagon‐like peptide 1 receptor agonists; LVEF, left ventricular ejection fraction; MI, myocardial infarction; PCI, percutaneous coronary intervention; SGLT2i, sodium‐dependent glucose transporters 2 inhibitors; STEMI, ST‐segment elevation myocardial infarction; TIA, transient ischemic attacks.

^a^ Continuous beta‐blocker therapy versus discontinuous beta‐blocker therapy (consisting of the beta‐blocker therapy <6 months and the no‐beta‐blocker therapy).

^b^ Continuous beta‐blocker therapy versus beta‐blocker therapy <6 months.

^c^ Continuous beta‐blocker therapy vs.versus no‐beta‐blocker therapy.

* *p* < 0.05

** *p* < 0.01

*** *p* < 0.001.

### 2.4 Definitions

The primary outcomes were cardiac death and major adverse cardiovascular events (MACE, composite endpoint event of cardiac death, rehospitalization for HF, recurrent MI). The secondary outcomes were all‐cause death, stroke, unstable angina, rehospitalization for HF, recurrent MI. Cardiac death was defined as death due to fatal MI, HF, and death that cannot be attributed to noncardiac causes. HF was defined as a previous history of HF or the presence of signs or symptoms associated with HF predischarge. Left ventricular systolic dysfunction was defined as LVEF below 40%. Continuous beta‐blocker therapy was defined as persistent treatment with beta‐blockers that lasted >6 months. Beta‐blocker therapy <6 months was defined as discharge prescription of beta‐blockers but lasting less than 6 months. No‐beta‐blocker therapy was described as never treated with beta‐blockers. Others were described as discharged without beta‐blocker therapy, restarted beta‐blocker therapy during the follow‐up. AMI is defined by the elevation of serum markers of myocardial injury at least twice their upper limit of normal (creatine kinase isoenzyme or troponin I), ST‐segment elevation or decrease in at least two contiguous leads greater than 0.1 mv, and pathological Q waves. LVEF is measured by the Simpson method of cardiac ultrasound, which is determined by the last measurement taken during hospitalization. Other PCI includes delayed PCI and rescue PCI. Timely reperfusion therapy was considered <12 h from symptom onset to PCI therapy, <90 min from door‐to‐balloon, and <30 min from first medical contact to thrombolytic therapy.

## 3 RESULTS

### 3.1 Clinical characteristics

Our study first analyzed the differences in clinical characteristics between patients in the continuous beta‐blocker therapy group and those in the discontinuous beta‐blocker therapy group, and then separately between the continuous beta‐blocker therapy group patients and the two subgroups of patients (the no‐beta‐blocker therapy group and the beta‐blocker therapy <6 months group).

Compared with patients treated with discontinuous beta‐blockers, patients treated with continuous beta‐blockers were younger (64.0 vs. 67.0 years, *p* = 0.005), had lower LVEF (57.0% vs. 57.0%, *p* = 0.025), had more combined hypertension (58.4% vs. 47.5%, *p* < 0.001) and cardiac aneurysm (4.7% vs. 2.2%, *p* = 0.043), had more anterior wall MI (61.1% vs. 33.3%, *p* < 0.001) and less inferior/posterior wall MI (42.6% vs.68.0%, *p* < 0.001), were more frequently treated with coronary angiography (94.8% vs. 90.4%, *p* = 0.005) and PCI (82.2% vs. 70.8%, *p* < 0.001), and more frequently treated with dual antiplatelet (94.7% vs. 91.3%, *p* = 0.029), statin (99.4% vs. 97.8%, *p* = 0.014), and ACEI/ARB/ARNI (72.5% vs. 54.2%, *p* < 0.001) medications. Detailed baseline characteristics were shown in Table 1. Baseline characteristics of continuous beta‐blocker therapy with both subgroups were also described in detail in Table 1.

For the present study, patients treated with continuous beta‐blockers accounted for 93.0% (1001/1076) of the included patients, and the proportion of patients treated with beta‐blockers for <6 months was 7.0% (75/1076). And, we obtained the reasons associated with 68 discontinuous patients (68/75, 90.7%) from healthcare data and telephone contacts, of which 65 were discontinued for their reasons (e.g., unawareness of the need for long‐term medication after MI, fear of adverse drug reactions, isolation for epidemic reasons, etc.) and 3 were discontinued due to new‐onset disease or slow heart rate.

### 3.2 Outcomes

**Figure 2:**
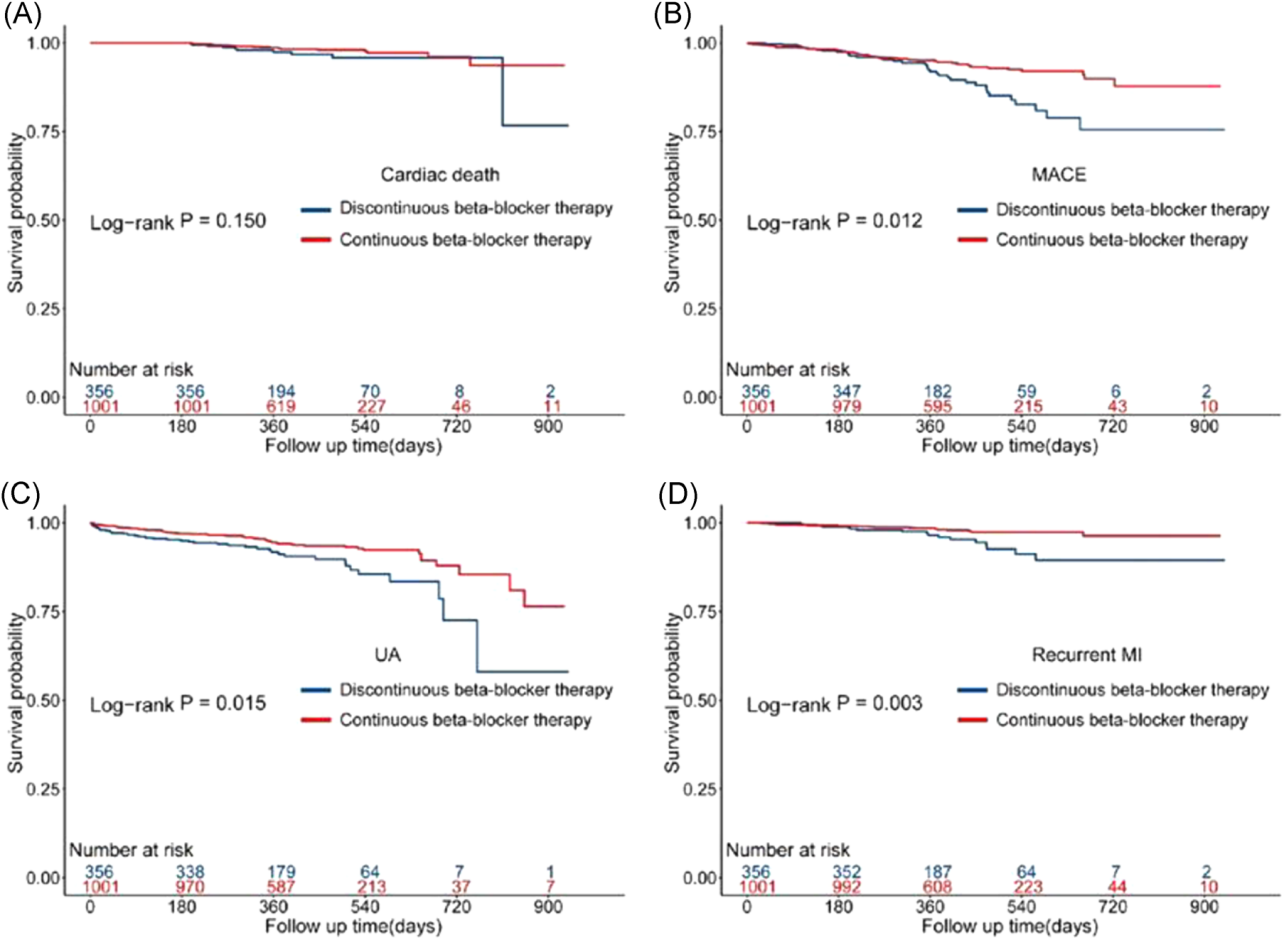
Kaplan–Meier survival estimates. This figure demonstrates the association between continuous beta‐blocker therapy and outcomes (including cardiac death, unstable angina, recurrent MI, mace). The population included patients with continuous beta‐blocker therapy (*N* = 1001) and patients with discontinuous beta‐blocker therapy (*N* = 356). A log‐rank test was used, uncorrected. MACE, major adverse cardiovascular events; MI, myocardial infarction

We followed the enrolled patients for a median of 13.0 (9.2–17.4) months at discharge. We first compared the outcomes of patients treated with continuous beta‐blockers with those treated with discontinuous beta‐blockers. The results suggested that continuous beta‐blocker therapy was associated with a reduced risk of unstable angina (IPTW correction, hazard ratio [HR]: 0.50; 95% CI: 0.32–0.79; *p* = 0.002), recurrent MI (IPTW correction, HR: 0.32; 95% CI: 0.16–0.66; *p* = 0.012), and MACE (IPTW correction, HR: 0.51; 95% CI: 0.32–0.82; *p* = 0.006), with or without IPTW correction. While there was no statistical correlation between continuous beta‐blocker therapy and the risk of cardiac death (Cox regression analyses, HR: 0.57; 95% CI: 0.26–1.24; *p* = 0.155), nor after IPTW adjusted (IPTW correction, HR: 0.57; 95% CI: 0.24–1.36; *p* = 0.206). Other outcomes, such as all‐cause death (IPTW correction, HR: 0.50; 95% CI: 0.23–1.07; *p* = 0.074), stroke (IPTW correction, HR: 0.44; 95% CI: 0.11–1.73; *p* = 0.243), and rehospitalization for HF (IPTW correction, HR: 0.75; 95% CI: 0.37–1.51; *p* = 0.420), showed no remarkable distinction between the two groups (Table 2). The Kaplan–Meier survival curves also suggested similar results (Figure 2).

**Table 2:** Risk of cardiovascular and cerebrovascular events

Events	Continuous beta‐blocker therapy (*N* = 1001)	Discontinuous beta‐blocker therapy (*N* = 356)	*p*‐value
*MACE*			
No. of patients with event	60/1001 (6.0%)	37/356 (10.4%)	‐
Unadjusted HR (95% CI)^a^	0.53 (0.35–0.80)	1.00 (ref)	0.013
Adjusted with IPTW (95% CI)^b^	0.51 (0.32–0.82)	1.00 (ref)	0.006
*Cardiac death*			
No. of patients with event	18/1001 (1.8%)	10/356 (2.8%)	‐
Unadjusted HR (95% CI)	0.57 (0.26–1.24)	1.00 (ref)	0.155
Adjusted with IPTW (95% CI)	0.57 (0.24–1.36)	1.00 (ref)	0.206
*Recurrent myocardial infarction*			
No. of patients with event	20/1001 (2.0%)	17/356 (4.8%)	‐
Unadjusted HR (95% CI)	0.39 (0.20–0.75)	1.00 (ref)	0.004
Adjusted with IPTW (95% CI)	0.32 (0.16–0.66)	1.00 (ref)	0.012
*Rehospitalization for heart failure*			
No. of patients with event	36/1001 (3.6%)	17/356 (4.8%)	‐
Unadjusted HR (95% CI)	0.707 (0.40–1.26)	1.00 (ref)	0.238
Adjusted with IPTW (95% CI)	0.75 (0.37–1.51)	1.00 (ref)	0.420
*Rehospitalization for unstable angina*			
No. of patients with event	63/1001 (6.3%)	37/356 (10.4%)	‐
Unadjusted HR (95% CI)	0.55 (0.36–0.82)	1.00 (ref)	0.015
Adjusted with IPTW (95% CI)	0.50 (0.32–0.79)	1.00 (ref)	0.002
*All‐cause death*			
No. of patients with event	21/1001 (2.1%)	13/356 (3.7%)	‐
Unadjusted HR (95% CI)	0.51 (0.26–1.02)	1.00 (ref)	0.058
Adjusted with IPTW (95% CI)	0.50 (0.23–1.07)	1.00 (ref)	0.074
*Stroke*			
No. of patients with event	9/1000 (0.9%)	5/356 (1.4%)	‐
Unadjusted HR (95% CI)	0.62 (0.21–1.84)	1.00 (ref)	0.387
Adjusted with IPTW (95% CI)	0.44 (0.11–1.73)	1.00 (ref)	0.243

Abbreviations: HR, hazard ratio; MACE, major adverse cardiovascular events; ref, reference.

^a^ Cox univariate analysis was used to analyze.

^b^ Correction was performed using inverse probability treatment weighting (IPTW), included variables were sex, age, LVEF, type of myocardial infarction, site of myocardial infarction (anterior MI; inferior/posterior MI; other sites MI), history of hypertension, history of diabetes mellitus, history of chronic kidney disease, history of coronary artery disease, history of stroke, family history of coronary artery disease, history of hyperlipidemia, history of smoking, history of tumor, history of atrial fibrillation, coronary angiography, PCI therapy, thrombolytic therapy, type of PCI, timely reperfusion therapy, total reperfusion therapy, coronary artery bypass grafting, cardiac aneurysm, anticoagulants, aspirin, clopidogrel/ticagrelor, statins, diuretics, ACEI/ARB/ARNI, SGLT2i/DPP4i/GLP1Ras.

In addition to the primary analysis between the two groups described above, we then compared the continuous beta‐blocker therapy group with the no‐beta‐blocker therapy group and the beta‐blocker‐treated <6 months group, respectively. The results suggested that continuous beta‐blocker therapy remained associated with a reduced risk of unstable angina, recurrent MI, and MACE. Each endpoint event is described in detail in Table S1, Figure S1.

From our study, continuous beta‐blocker therapy was associated with improved outcomes, and the long‐term application of beta‐blockers (≥6 months) may be superior to the short‐term application of beta‐blockers (<6 months).

### 3.3 Subgroups analysis

**Figure 3:**
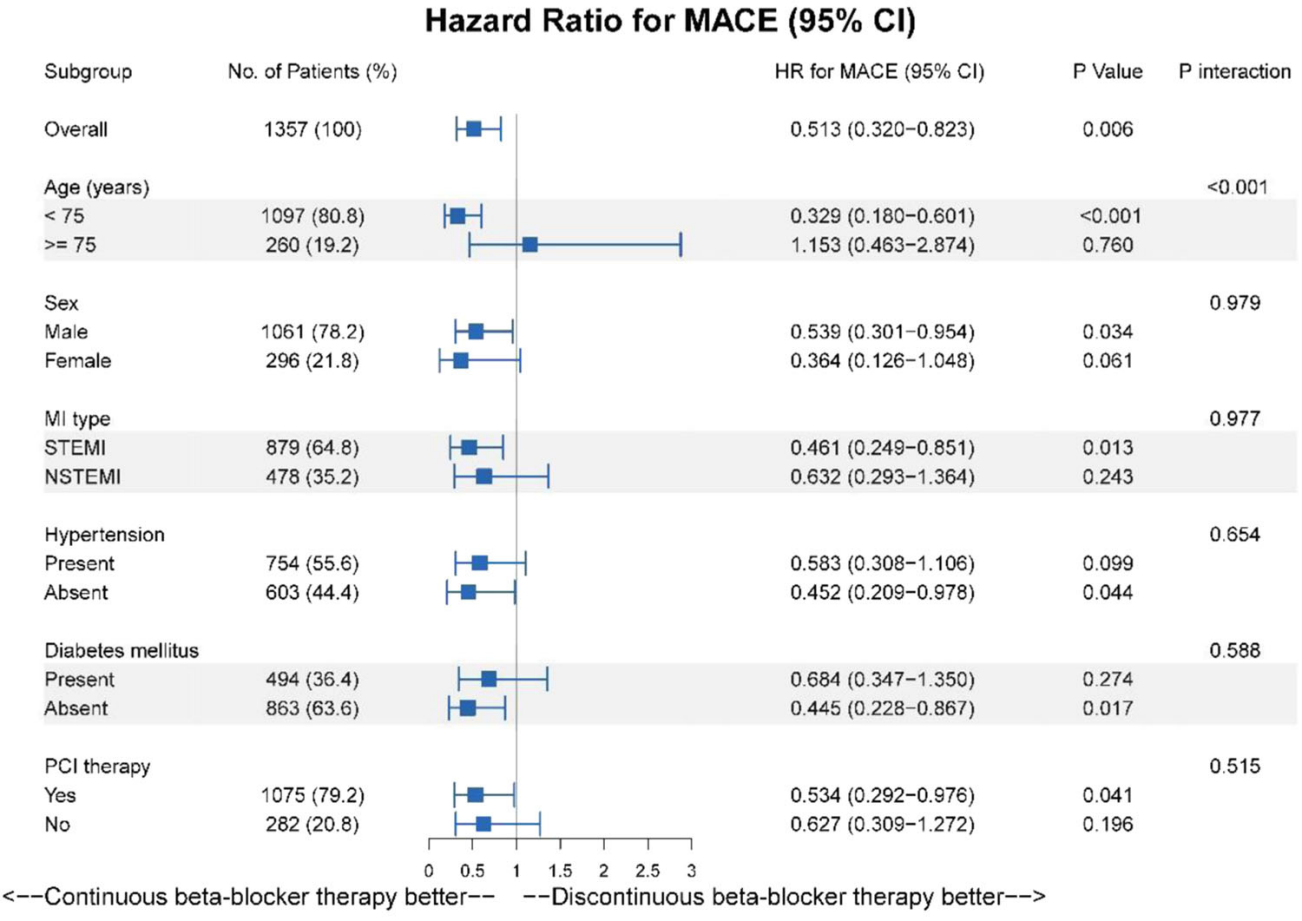
Subgroups analysis. The associations of beta‐blocker therapy with major adverse cardiovascular events (MACE) were analyzed in different subgroups. The population included patients with continuous beta‐blocker therapy (*N* = 1001) and patients with discontinuous beta‐blocker therapy (*N* = 356). The *p*‐values were adjusted with propensity score inverse probability treatment weighting, and the adjusted factors are shown in Table 2. HR, hazard ratio; MI, myocardial infarction; PCI, percutaneous coronary intervention; STEMI, ST‐segment elevation myocardial infarction

This study performed a subgroup analysis for the risk of MACE, with the population consisting of patients treated with continuous beta‐blockers and patients treated with discontinuous beta‐blockers. Subgroup analyses were conducted by age (age <75 years vs. ≥75 years), sex, type of MI (STEMI vs. NSTEMI), hypertension, diabetes, and PCI therapy. Based on propensity scores with IPTW, the results suggested a statistically significant association between continuous beta‐blocker therapy and reduced risk of MACE in the subgroups of patients aged <75 years, male patients, STEMI, absence of hypertension, absence of diabetes, treatment with PCI (Figure 3).

### 3.4 Sensitivity analysis

There is a sizable difference in the number of patients in the two groups of continuous beta‐blocker therapy (*N* = 1001) and beta‐blocker therapy <6 months (*N* = 75). We performed a sensitivity analysis using propensity score matching to test the relationships between continuous beta‐blocker therapy and outcomes. We performed logit regression with prescribed continuous beta‐blocker therapy as the dependent variable and each variable in Table 1 as a covariate (method, nearest; ratio, 4:1; caliper, 0.02). The study was successful in matching 299 patients (continuous beta‐blocker therapy, *N* = 233; beta‐blocker therapy <6 months, *N* = 66). The results showed continuous beta‐blocker therapy was also associated with a reduced risk of unstable angina or MACE after IPTW correction. However, there was no significant association with the risk of recurrent MI. The association of continuous beta‐blocker therapy with all outcomes was shown in Table S2.

## 4 DISCUSSION

In this prospective, multicenter, observational study, we found a statistically significant difference between continuous beta‐blocker therapy and a reduced risk of unstable angina, recurrent MI, and MACE in patients without HF or left ventricular systolic dysfunction after AMI, and, importantly, the duration of beta‐blocker therapy is preferable to long‐term (≥6 months). The association between continuous beta‐blocker therapy and cardiac death was not observed in our study. The beneficial effects of continuous beta‐blocker therapy were presented in several subgroups.

A considerable number of studies exist that assess the relationship between beta‐blocker therapy and clinical outcomes in patients with MI. However, most studies have explored the relationship between the use of beta‐blockers at a particular time point and outcomes or the long‐term use of beta‐blockers and outcomes through a comparison of the clinical outcomes of patients treated or not treated with beta‐blockers. The results of their studies are also inconsistent.^12^, ^13^, ^14^, ^15^ Concerning the duration of beta‐blocker therapy, as mentioned previously, the latest ESC guidelines did not clearly state the specific duration of beta‐blocker use in patients with AMI. In reality, due to ethical review and other factors (a small percentage of patients discontinuing beta‐blockers implies a small sample size^16^, ^17^, ^18^), randomization of beta‐blocker use or duration would be difficult to achieve. Only a very few observational studies have currently investigated the issue of the duration of beta‐blocker therapy.

A retrospective, national, cohort study (*N* = 28,970, median follow‐up 3.5 years) in patients without HF (defined as previous HF) after AMI, including a beta‐blocker therapy <1‐year group and a beta‐blocker therapy ≥1‐year group, suggested that continued beta‐blocker therapy ≥1 year after MI is associated with a reduced risk of all‐cause death and a reduced risk of composite outcomes (a composite of all‐cause death, recurrent MI, or hospitalization for new HF). As mentioned by the authors of the study, no information on LVEF was included. This cohort included patients with left ventricular systolic dysfunction (LVEF < 40%) who might have a worse prognosis despite being treated with beta‐blockers for ≥1 year than those without left ventricular systolic dysfunction but treated with beta‐blockers for <1 year.^19^

Similarly, another large‐scale cohort study (*N* = 73,450, median follow‐up 3.8 years), designed to explore the effects of stopping beta‐blockers in patients without HF after AMI, divided the patients according to beta‐blocker use, and the results suggested that discontinuation of beta‐blockers beyond 1 year was related to an increased risk of all‐cause death or readmission for the acute coronary syndrome, while statistical significance was not reached for the association with all‐cause death. Regulatory information on LVEF was also unfortunately not available for this study. In addition, the findings of this study cannot be generalized to the first year because follow‐up began 1 year after the AMI index.^18^

Both of the above studies examined differences in outcomes in patients treated with beta‐blockers for ≥1 year versus those treated with beta‐blockers for <1 year, and both suggest that long‐term treatment with beta‐blockers might be beneficial in patients without HF after AMI, although not both suggested improvement in all‐cause death. LVEF < 40% or LVEF ≥ 40% is an indispensable criterion for assessing beta‐blocker therapy as recommended by the latest ESC Guidelines in patients with AMI without HF.^5^ Our study focused on the shorter duration of discontinuation of beta‐blocker therapy (<6 months) and included information on LVEF. The results suggest a statistically significant association between continuous beta‐blocker therapy (≥6 months) and better outcomes. Beta‐blocker therapy should probably be longer than 6 months in patients without HF or left ventricular systolic dysfunction after AMI. The present study might be able to add to the results of the large‐scale cohort study described above.

In our study, a lower proportion of no‐beta‐blocker therapy patients underwent PCI, which may be explained by a greater proportion of such patients being older than 75 years, a greater proportion with previous comorbid CAD, a greater incidence of inferior/posterior MI, and more unstable blood pressure, resulting in a lower willingness to undergo PCI, poorer revascularization, and less prescription of beta‐blockers and ACEI/ARB/ARNI.

### 4.1 Limitations

Our research has limitations. First, our study is a small observational study, the scientific validity of the study is limited by the sample size and the inherent failure to correct for unknown additional confounders (such as economic income, education level, and results of coronary angiography). Second, we lost information on the dose of beta‐blockers used in a larger number of patients during follow‐up, and we had no way to confirm whether patients treated with beta‐blockers were receiving the optimal dose. The association between beta‐blocker dose and outcomes could not be assessed.

## 5 CONCLUSIONS

Continuous beta‐blocker therapy was not statistically associated with cardiac death; yet, continuous beta‐blocker therapy was associated with a reduced risk of unstable angina or recurrent MI or MACE in patients without HF or left ventricular systolic dysfunction after AMI, and could be better with long‐term therapy (≥6 months).

## CONFLICTS OF INTEREST

The authors declare no conflicts of interest.

## AUTHOR CONTRIBUTIONS

Xue‐Song Wen participated in the design of the registry, collected the data, performed the statistical analysis, and drafted the manuscript. Rui Luo and Qin Duan were involved in data collection. Shu Qin, Jun Xiao, and Dong‐Ying Zhang were responsible for the study concept, design, and final approval of the manuscript. Xue‐Song Wen is the first author. All authors have read and approved the final manuscript.

## Supporting information


**Table S1. Risk of cardiovascular and cerebrovascular events in subgroups. Table S2. Risk of cardiovascular and cerebrovascular events in patients after propensity score matching (PSM)** ^**a**^ **Figure S1. Kaplan‐Meier Survival Estimates**.

## Data Availability

The datasets used and/or analyzed during the current study are available from the corresponding author on reasonable request.
