# Supplementary material for: The duration of beta‐blocker therapy and outcomes in patients without heart failure or left ventricular systolic dysfunction after acute myocardial infarction: A multicenter prospective cohort study
Source: Clin Cardiol. 2022 Mar 4;45(5):509–18. doi: 10.1002/clc.23807 (PMC9045069; doi:10.1002/clc.23807)
Supplement: Supplementary file 1 — Table S1. Risk of cardiovascular and cerebrovascular events in subgroups. Table S2. Risk of cardiovascular and cerebrovascular events in patients after propensity score matching (PSM) a Figure S1. Kaplan‐Meier Survival Estimates. [file CLC-45-509-s001.docx]

**Table S1. Risk of cardiovascular and cerebrovascular events** **in subgroups.**

| **Events** | **Duration** | | | **With or without** | | |
| --- | --- | --- | --- | --- | --- | --- |
|  | Continuous beta-blocker therapy  (N=1001) | Beta-blocker therapy <6 months (N=75) | *P*-value | Continuous beta-blocker therapy  (N=1001) | No-beta-blocker therapy  (N=281) | *P*-value |
|  |  |  |  |  |  |  |
| **MACE** | | | | | | |
| No. of patients with event | 60/1001 (6.0%) | 9/75 (12.0%) |  | 60/1001 (6.0%) | 28/281 (10.0%) |  |
| Unadjusted HR (95% CI) **^a^** | 0.43 (0.21-0.87) | 1.00 (ref) | 0.019 | 0.57 (0.36-0.89) | 1.00 (ref) | 0.014 |
| Adjusted with IPTW (95%CI) **^b^** | 0.44 (0.19-0.94) | 1.00 (ref) | 0.047 | 0.58 (0.34-0.97) | 1.00 (ref) | 0.036 |
| **Cardiac death** | | | | | | |
| No. of patients with event | 18/1001 (1.8%) | 2/75 (2.7%) |  | 18/1001 (1.8%) | 8/281 (2.8%) |  |
| Unadjusted HR (95% CI) | 0.54 (0.13-2.36) | 1.00 (ref) | 0.415 | 0.58 (0.25-1.33) | 1.00 (ref) | 0.196 |
| Adjusted with IPTW (95% CI) | 1.05 (0.23-4.76) | 1.00 (ref) | 0.950 | 0.62 (0.25-1.51) | 1.00 (ref) | 0.291 |
| **Rehospitalization for heart failure** | | | | | | |
| No. of patients with event | 36/1001 (3.6%) | 5/75 (6.7%) |  | 36/1001 (3.6%) | 12/281 (4.3%) |  |
| Unadjusted HR (95% CI) | 0.47 (0.18-1.20) | 1.00 (ref) | 0.116 | 0.81 (0.42-1.56) | 1.00 (ref) | 0.525 |
| Adjusted with IPTW (95% CI) | 0.69 (0.24-2.02) | 1.00 (ref) | 0.502 | 0.75 (0.34-1.66) | 1.00 (ref) | 0.474 |
| **Recurrent myocardial infarction** | | | | | | |
| No. of patients with event | 20/1001 (2.0%) | 4/75 (5.3%) |  | 20/1001 (2.0%) | 13/281 (4.6%) |  |
| Unadjusted HR (95% CI) | 0.33 (0.11-0.96) | 1.00 (ref) | 0.042 | 0.41 (0.21-0.83) | 1.00 (ref) | 0.013 |
| Adjusted with IPTW (95% CI) | 0.25 (0.08-0.79) | 1.00 (ref) | 0.019 | 0.42 (0.20-0.89) | 1.00 (ref) | 0.024 |
| **Rehospitalization for unstable angina** | | | | | | |
| No. of patients with event | 63/1001 (6.3%) | 9/75 (12.0%) |  | 63/1001 (6.3%) | 28/281 (10.0%) |  |
| Unadjusted HR (95% CI) | 0.44 (0.22-0.89) | 1.00 (ref) | 0.023 | 0.58 (0.37-0.91) | 1.00 (ref) | 0.017 |
| Adjusted with IPTW (95% CI) | 0.42 (0.19-0.91) | 1.00 (ref) | 0.028 | 0.54 (0.33-0.89) | 1.00 (ref) | 0.016 |
| **All-cause death** | | | | | | |
| No. of patients with event | 21/1001 (2.1%) | 3/75 (4.0%) |  | 21/1001 (2.1%) | 10/281 (3.6%) |  |
| Unadjusted HR (95% CI) | 0.41 (0.12-1.40) | 1.00 (ref) | 0.156 | 0.54 (0.26-1.15) | 1.00 (ref) | 0.111 |
| Adjusted with IPTW(95% CI) | 0.67 (0.19-2.39) | 1.00 (ref) | 0.540 | 0.54 (0.24-1.24) | 1.00 (ref) | 0.145 |
| **Stroke** | | | | | | |
| No. of patients with event | 9/1000 (0.9%) | 1/75 (1.3%) |  | 9/1000 (0.9%) | 4/281 (1.4%) |  |
| Unadjusted HR (95% CI) | 0.61 (0.08-4.84) | 1.00 (ref) | 0.641 | 0.61 (0.19-1.99) | 1.00 (ref) | 0.416 |
| Adjusted with IPTW (95% CI) | 1.41 (0.18-10.98) | 1.00 (ref) | 0.741 | 0.62 (0.17-2.22) | 1.00 (ref) | 0.458 |

Abbreviations: MACE, major adverse cardiovascular events (composite endpoint event of cardiac death, new-onset rehospitalization for HF, recurrent myocardial infarction), HR, hazard ratio, ref, reference.

**^a^** Cox univariate analysis was used to analyze.

**^b^** Correction was performed using inverse probability treatment weighting (IPTW), included variables were sex, age, LVEF, type of myocardial infarction, site of myocardial infarction (anterior MI; inferior/posterior MI; other sites MI), history of hypertension, history of diabetes mellitus, history of chronic kidney disease, history of coronary artery disease, history of stroke, family history of coronary artery disease, history of hyperlipidemia, history of smoking, history of tumor, history of atrial fibrillation, coronary angiography, PCI therapy, thrombolytic therapy, type of PCI, timely reperfusion therapy, total reperfusion therapy, coronary artery bypass grafting, cardiac aneurysm, anticoagulants, aspirin, clopidogrel/ticagrelor, statins, diuretics, ACEI/ARB/ARNI, SGLT2i/DPP4i/GLP1Ras.

**Table S2. Risk of cardiovascular and cerebrovascular events in patients after propensity score matching (PSM) ^a^.**

| **Events** | Continuous beta-blocker therapy (N=233) | Beta-blocker therapy <6 months (N=66) | *P-value* |
| --- | --- | --- | --- |
| **MACE** | | | |
| No. of patients with event | 18/233 (7.7%) | 7/66 (10.6%) |  |
| Unadjusted HR (95% CI) **^b^** | 0.59 (0.37-0.87) | 1.00 (ref) | 0.032 |
| Adjusted with IPTW (95% CI) **^c^** | 0.54 (0.40-0.92) | 1.00 (ref) | 0.046 |
| **Cardiac death** | | | |
| No. of patients with event | 6/233 (2.6%) | 1/66 (1.5%) |  |
| Unadjusted HR (95% CI) | 1.24 (0.15-10.36) | 1.00 (ref) | 0.844 |
| Adjusted with IPTW (95% CI) | 1.44 (0.16-12.65) | 1.00 (ref) | 0.742 |
| **Recurrent myocardial infarction** | | | |
| No. of patients with event | 5/233 (2.1%) | 4/66 (6.1%) |  |
| Unadjusted HR (95% CI) | 0.27 (0.07-1.02) | 1.00 (ref) | 0.054 |
| Adjusted with IPTW (95% CI) | 0.30 (0.08-1.19) | 1.00 (ref) | 0.087 |
| **Rehospitalization for heart failure** | | | |
| No. of patients with event | 12/233 (5.4%) | 3/66 (5.5%) |  |
| Unadjusted HR (95% CI) | 0.89 (0.25-3.16) | 1.00 (ref) | 0.853 |
| Adjusted with IPTW (95% CI) | 0.80 (0.23-2.83) | 1.00 (ref) | 0.729 |
| **Rehospitalization for unstable angina** | | | |
| No. of patients with event | 13/233 (5.6%) | 9/66 (13.6%) |  |
| Unadjusted HR (95% CI) | 0.33 (0.14-0.80) | 1.00 (ref) | 0.014 |
| Adjusted with IPTW (95% CI) | 0.37 (0.15-0.93) | 1.00 (ref) | 0.035 |
| **All-cause death** | | | |
| No. of patients with event | 6/233(2.6%) | 2/66 (3.0%) |  |
| Unadjusted HR (95% CI) | 0.62 (0.12-3.11) | 1.00 (ref) | 0.563 |
| Adjusted with IPTW (95% CI) ^b^ | 0.64 (0.13-3.22) | 1.00 (ref) | 0.585 |
| **Stroke** | | | |
| No. of patients with event | 1/232 (0.4%) | 1/66 (1.5%) |  |
| Unadjusted HR (95% CI) | 0.28 (0.02-4.51) | 1.00 (ref) | 0.371 |
| Adjusted with IPTW (95% CI) | 0.30 (0.02-4.69) | 1.00 (ref) | 0.389 |

**^a^** Continuous beta-blocker therapy (after PSM, N=233); Beta-blocker therapy <6 months (after PSM, N=66).

Abbreviations: MACE, major adverse cardiovascular events, HR, hazard ratio, ref, reference.

**^b^** Cox univariate analysis was used to analyze.

**^c^** Correction was performed using inverse probability treatment weighting (IPTW), included variables were sex, age, LVEF, type of myocardial infarction, site of myocardial infarction (anterior MI; inferior/posterior MI; other sites MI), history of hypertension, history of diabetes mellitus, history of chronic kidney disease, history of coronary artery disease, history of stroke, family history of coronary artery disease, history of hyperlipidemia, history of smoking, history of tumor, history of atrial fibrillation, coronary angiography, PCI therapy, thrombolytic therapy, type of PCI, timely reperfusion therapy, total reperfusion therapy, coronary artery bypass grafting, cardiac aneurysm, anticoagulants, aspirin, clopidogrel/ticagrelor, statins, diuretics, ACEI/ARB/ARNI, SGLT2i/DPP4i/GLP1Ras.

**Figure S1. Kaplan-Meier Survival Estimates.**

**Figure S1** shows
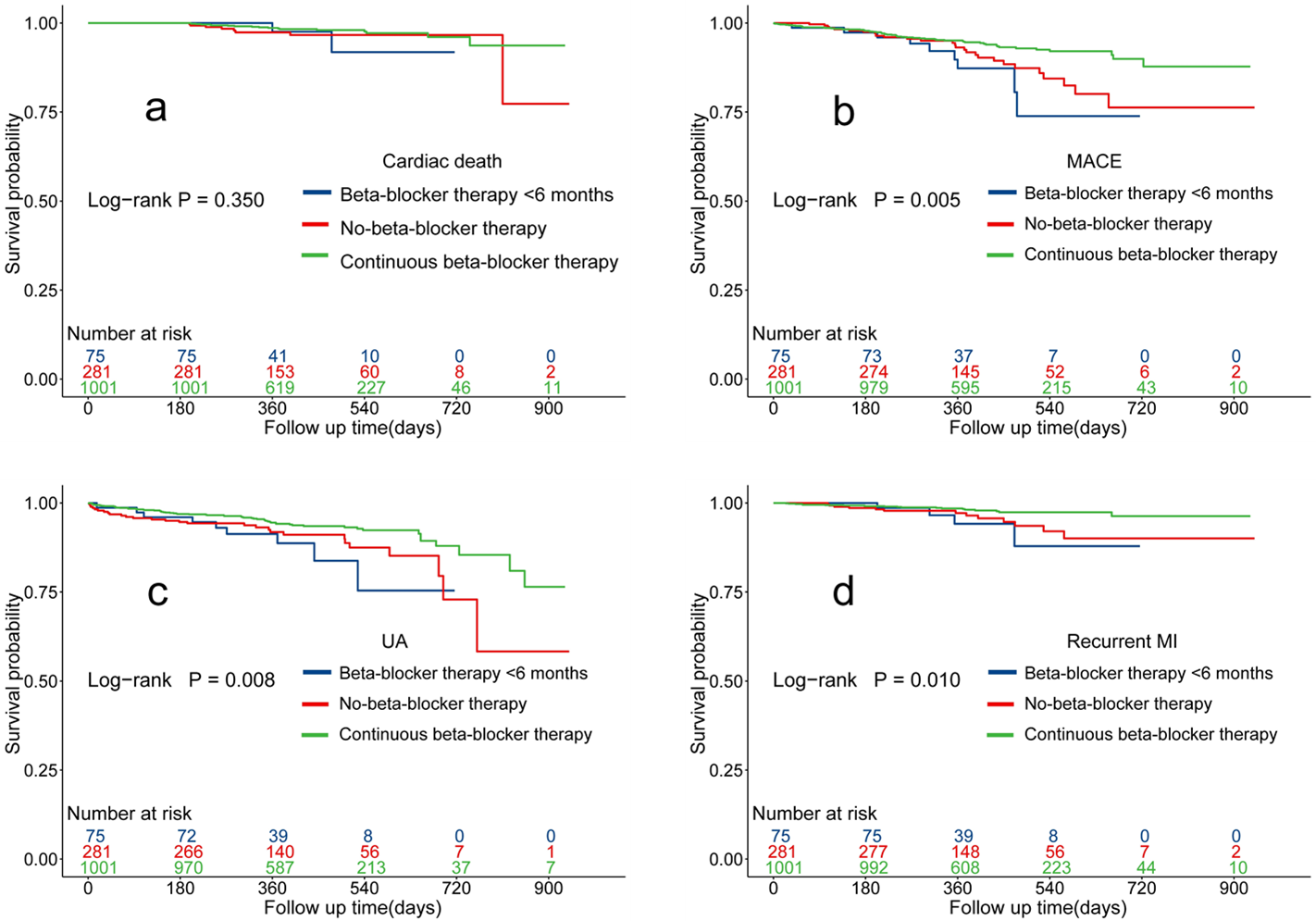
the association between beta-blocker therapy and outcomes (including all-cause death, unstable angina, recurrent myocardial infarction, mace). the population included patients with continuous beta-blocker therapy (N=1001), patients with no-beta-blocker therapy (N=281), and patients with beta-blocker therapy <6 months (N=75). A log-rank test was used, uncorrected.
